# Do physicians with academic affiliation have lower burnout and higher career-related satisfaction?

**DOI:** 10.1186/s12909-022-03327-5

**Published:** 2022-04-26

**Authors:** Chu Zhuang, Xiaochu Hu, Michael J. Dill

**Affiliations:** 1 Department of Health Policy and Management, University of Maryland, College Park, School of Public Health, Maryland, 20742 USA; 2 Association of American Medical Colleges, 655 K St. NW Suite 100, Washington DC, USA

**Keywords:** Medical education, Burnout, Career-related satisfaction, Faculty, Teaching

## Abstract

**Background:**

Physicians report increasing burnout and declining career-related satisfaction, negatively impacting physician well-being and patient care quality. For physicians with academic affiliations, these issues can directly affect future generations of physicians. Previous research on burnout and satisfaction has focused on factors like work hours, gender, race, specialty, and work setting. We seek to contribute to the literature by examining these associations while controlling for demographic, family, and work-related characteristics. Furthermore, we aim to determine any differential effects of faculty rank.

**Methods:**

We analyzed data on practicing physicians in the U.S. from the Association of American Medical College’s (AAMC) 2019 National Sample Survey of Physicians (NSSP,) which includes variables adapted from the Maslach Burnout Inventory. We used ordinal logistic regressions to explore associations between academic affiliation and burnout. We conducted a factor analysis to consolidate satisfaction measures, then examined their relationship with academic affiliation using multivariate linear regressions. All regression analyses controlled for physicians’ individual, family, and work characteristics.

**Results:**

Among respondents (*n* = 6,000), 40% were affiliated with academic institutions. Physicians with academic affiliations had lower odds than their non-affiliated peers for feeling emotional exhaustion every day (Odds Ratio [OR] 0.87; 95% CI: 0.79–0.96; *P* < *.001)* and reported greater career-related satisfaction (0.10–0.14, SE, 0.03, 0.02; *P* < *.001*). The odds of feeling burnt out every day were higher for associate professors, (OR 1.57; 95% CI: 1.22–2.04; *P* < *.001*) assistant professors, (OR 1.64; 95% CI: 1.28–2.11; *P* < *.001*), and instructors (OR 1.72; 95% CI, 1.29–2.29; *P* < *.001*), relative to full professors.

**Conclusions:**

Our findings contribute to the literature on burnout and career satisfaction by exploring their association with academic affiliation and examining how they vary among different faculty ranks. An academic affiliation may be an essential factor in keeping physicians’ burnout levels lower and career satisfaction higher. It also suggests that policies addressing physician well-being are not “one size fits all” and should consider factors such as academic affiliation, faculty rank and career stage, gender identity, the diversity of available professional opportunities, and institutional and social supports. For instance, department chairs and administrators in medical institutions could protect physicians’ time for academic activities like teaching to help keep burnout lower and career satisfaction higher.

**Supplementary Information:**

The online version contains supplementary material available at 10.1186/s12909-022-03327-5.

## Background

Physician well-being is a complex, multifactorial issue impacting the nation’s health care system [1]. Physician burnout, characterized by emotional exhaustion, depersonalization, and feelings of low personal accomplishment at work, has become ubiquitous, raising concerns for well-being, patient safety, and quality of care [1–3]. At the same time, physicians’ career-related satisfaction is on the decline, adding further strain to physicians’ mental health, professional relationships with colleagues, and relationship with patients [4, 5]. For example, studies of U.S. physicians from 2011 to 2014 found an increase in burnout (46% vs. 55%) and a decrease in career satisfaction over time (49% vs. 41%) [5, 6].

These issues may carry more weight for physicians with academic affiliations (faculty). The U.S. has 192 accredited medical schools and 400 teaching hospitals, each of which relies on physician faculty members to teach and prepare the next generation of physicians. Although physicians with academic affiliations influence the future physician workforce, only a few studies have examined their experiences with burnout and career-related satisfaction. Dandar et al.’s (2019) study of burnout by medical school faculty rank, the only such study of its kind, found that associate professors reported higher levels of burnout than assistant or full professors [7]. A 2018 survey of physicians from just one specialty, U.S. academic radiologists, found that 79% of respondents reported experiencing burnout at least once [8]. Surveying academic faculty across specialties, del Carmen et al. (2019) reported 46.5% of respondents experienced burnout in 2017, compared to 40.6% in 2014 [9]. Studies found that burnout was higher for early career faculty compared to mid- to late-career faculty [10, 11]. Similarly, research indicates that physicians’ career-related satisfaction varies by several factors, including career stage [10–12]. However, these studies offer no comparisons between physicians with and without academic affiliation. Thus, the relationship between academic affiliation and burnout and career satisfaction is an under-studied topic. We seek to contribute to the literature by examining these associations while controlling for demographic, family, and work-related characteristics. Furthermore, we aim to determine any differential effects of faculty rank. This knowledge is essential to understanding and improving physician well-being.

## Methods

### Dataset

We used data from the Association of American Medical Colleges’ (AAMC) National Sample Survey of Physicians (NSSP), developed in part by two authors on this paper. The NSSP is a large survey intended to inform multiple research projects. Survey data comprise individual- level characteristics, including measures of career-related satisfaction and select burnout measures from the Maslach Burnout Inventory. Referencing the American Medical Association’s (AMA) Physician Characteristics database [13], AAMC researchers set minimum numbers of responses for each of twenty-four age-sex-specialty group sampling strata based on the representation of each stratum within the study population, to achieve an overall sample of 6,000 physicians. A representative subset of the total population of active practicing physicians (*N* = 86,951) were invited to participate in the online survey in February 2019, and the survey closed in March 2019 once the targeted sample size had been achieved. After the survey, a set of analytical weights, based on the AMA data, were created using a combination of cell and rim weighting. The final weighted data thus match study population values for age group, sex, specialty group, and international medical graduates (IMGs). For details of the survey method, response rate, and post-survey analysis, see Additional file Sect. 1 and Additional file Table 1. A flow chart that describes the entire method process is presented in Additional file Fig. 1.

### Burnout and career-related satisfaction measures

To assess burnout, the NSSP included two items, emotional exhaustion and depersonalization, adapted from the Maslach Burnout Inventory (MBI) [2]. Among the full 22 MBI items, emotional exhaustion and depersonalization each represent a unique aspect of burnout and the two single-item measures exhibit strong, consistent associations with other items on the MBI [2, 14]. Given this research, combined with the fact that the NSSP was already a long survey, we felt confident using these two items to assess burnout without unduly burdening respondents. Specifically, respondents were provided the following statements, “*I feel more burned out from my work” (emotional exhaustion)* and *“I have become more callous toward people since I took this job” (depersonalization)*, then asked to report how often those statements reflect how they feel about practicing as a physician: “never,” “a few times a year or less,” “once a month or less,” “once a week,” “a few times a week,” and “every day.”

NSSP measured satisfaction by asking respondents to rank their satisfaction with the following seven elements using a 5-point Likert scale (“very dissatisfied”, “somewhat dissatisfied”, “neither satisfied or dissatisfied”, “somewhat satisfied”, “very satisfied”): career in medicine, specialty choice, income, amount of time spent interacting with each patient, the overall amount of time spent interacting with patients, the quality of time spent with patients, and ability to balance work and personal life. Additionally, using a 5-point Likert scale (“very unlikely", "somewhat unlikely”, “neither likely nor unlikely”, “somewhat likely”, “very likely”) participants answered the question, “*If you could do it all over again, how likely is it you would still want to be a doctor?”* Given correlations among the eight satisfaction measures, we sought to perform a factor analysis to extract fewer hypothetical satisfaction measures (factors).The consolidated factors are used in regression analysis as dependent variables and are more robust than any of the original measures.

### Academic Affiliation and Other Covariates

Our key independent variable, academic affiliation, is defined as “currently holding any type of faculty appointment at a medical school (including paid or volunteer, full-time or part-time).”

We included the following covariates in our models: gender, race/ethnicity, sexual orientation (heterosexual, or other, which includes gay or lesbian, bisexual, and self-identified ‘other’), age (grouped), rurality of the place they grew up (rural and non-rural), practice location (metropolitan area and nonmetropolitan area), marital status (married/partnered and non-married/non-partnered), specialty group, typical weekly work hours, IMG status (IMG and non-IMG.) Since a few studies have discussed how the various responsibilities of managing clinical duties alongside teaching and administrative duties may contribute to high levels of burnout reported by academic faculty [9, 11, 15], we controlled for weekly time spent in teaching (%).

We used three categories to describe participants’ race and ethnicity: Asian, White, and Under-represented in Medicine (URM). In our analyses, URM includes individuals identifying as American Indian or Alaska Native, Native Hawaiian or Other Pacific Islander, Black or African American, Hispanic/Latino or of Spanish Origin, and Other/Multiple races. Finally, we consolidated physicians’ practice specialties into four specialty groups: primary care specialties, medical specialties, surgical specialties, and other specialties.^1^

### Statistical method

Because respondents reported emotional exhaustion and depersonalization on a series of frequency options with uneven distances between adjacent levels, we performed ordinal logistic regressions to examine their associations with academic affiliation. Since 5-point Likert scales are standardized with equal distancing between adjacent levels, we ran multivariate linear regressions to determine the relationship between academic affiliation and satisfaction. We controlled for weights in multivariate linear regression analyses.^2^ We performed all statistical analyses in Stata/ SE 14.1 software. All methods were performed in accordance with the relevant guidelines and regulations.

## Results

### Descriptive statistics

The sample was 65% male and 69% white. Approximately 40% of the respondents were academically affiliated.^3^ Physicians with academic affiliations varied in their faculty rank: professor (13%), associate professor (27%), assistant professor (40%), instructor (16%), and other (adjunct professor/faculty, clinical/administrative staff) (4%). Additional file 1 displays detailed descriptive statistics for all independent variables (overall and by academic affiliation status). Distribution of burnout measures are presented in Table 1.

**Table 1:** Distribution of Burnout Measures

Frequency experienced	Emotional exhaustion *N* = 5,981 (%)	Depersonalization *N* = 5,969 (%)
Every day	8	5
A few times a week	17	10
Once a week	15	12
Once a month or less	20	14
A few times a year or less	28	25
Never	12	34
Total	100	100

Source: National Sample Survey of Physicians (2019), Association of American Medical Colleges

### Factor analysis

Factor analysis of the eight satisfaction measures suggested two unique satisfaction factors with an eigenvalue larger than 1: Time Use Satisfaction (eigenvalue of 4.26) and Career Satisfaction (eigenvalue of 1.21). Time Use Satisfaction included satisfaction with the amount of time spent interacting with each patient, the overall amount of time spent interacting with patients, the quality of time spent with patients, and the ability to balance work and personal life. Career satisfaction included the respondent’s satisfaction with their career in medicine, their specialty choice, their income, and the likelihood that they would still want to become a doctor if given a chance. (See Additional file 1 for factor loading results). We averaged the individual satisfaction measures to create the two new factors’ value and used them as dependent variables in the multivariable linear regressions. These consolidated satisfaction factors can be treated as continuous variables ranging from 1 (lowest satisfaction) to 5 (highest satisfaction.)

### Academic affiliation and burnout, career-related satisfaction

Table 2 shows the results of the ordinal logistic regression and multivariable linear regressions for academic affiliation and burnout and satisfaction. The dependent variables for each column are emotional exhaustion, depersonalization, time use satisfaction, and career satisfaction.

**Table 2:** Physician Burnout and Satisfaction, Controlling for Demographic and Practice Characteristics

	**Ordinal Logistic Regressions**		**Multivariable Linear Regressions**	
Independent Variables	Emotional Exhaustion^a^ (OR, 95% CI)	Depersonalization^a^ (OR, 95% CI)	Time Use Satisfaction (SE)	Career Satisfaction (SE)
Physicians with Academic Affiliation	0.87*** (0.79–0.96)	0.86*** (0.78–0.94)	0.11*** (0.03)	0.14*** (0.02)
Male	0.71*** (0.64–0.79)	0.87** (0.78–0.97)	0.15*** (0.03)	0.05* (0.02)
Under-represented in Medicine (URM)	0.80 (0.62–1.03)	0.82 (0.63–1.05)	0.03 (0.07)	0.09 (0.06)
Asian	0.92 (0.73–1.16)	0.85 (0.67–1.07)	0.09 (0.07)	0.09 (0.05)
White	1.04 (0.84–1.03)	1.04 (0.83–1.30)	0.01 (0.07)	0.07 (0.05)
Heterosexual/ Straight	0.93 (0.74–1.19)	0.84 (0.66–1.07)	0.07 (0.07)	0.04 (0.06)
Age < 35	Ref	Ref	Ref	Ref
Age [35, 55)	1.34** (1.08–1.67)	1.16 (0.93–1.46)	-0.26*** (0.07)	-0.11* (0.06)
Age [55,75)	1.27 (0.95–1.71)	0.78 (0.59–1.06)	-0.19* (0.09)	-0.01 (0.07)
Age ≥ 75	0.64 (0.38–1.12)	0.43** (0.24–0.77)	-0.05 (0.16)	0.08 (0.13)
Origin (Rural)	0.98 (0.86–1.11)	1.13 (0.99–1.29)	-0.02 (0.04)	0.08** (0.03)
Practice location: Metropolitan^b^	1.19 (0.96–1.45)	1.25* (1.01–1.53)	0.03 (0.06)	0.05 (0.05)
Married/Committed relationship	0.97 (0.85–1.11)	0.87* (0.77–0.99)	0.08* (0.04)	0.15*** (0.03)
Total number of children under 5^c^	0.88** (0.80–0.97)	0.99 (0.90–1.10)	-0.02 (0.03)	-0.03 (0.02)
Hours worked (per week)	1.01*** (1.01- 1.02)	1.01*** (1.00–1.01)	-0.01*** (0.00)	0.00*** (0.00)
Practice experience (years)	0.98*** (0.97–0.99)	0.98*** (0.97–0.99)	0.01*** (0.00)	0.01*** (0.00)
Specialty: Medical Specialties	Ref	Ref	Ref	Ref
Specialty: Other	1.13 (0.99–1.32)	1.11 (0.99–1.32)	0.02 (0.04)	-0.07* (0.03)
Specialty: Primary Care	1.33*** (1.17–1.52)	1.17* (1.03–1.34)	-0.12*** (0.09)	-0.19*** (0.03)
Specialty: Surgery	0.94 (0.82–1.10)	0.96 (0.83–1.13)	0.00 (0.11)	-0.03 (0.04)
International Medical Graduates (IMGs)	0.72*** (0.63–0.82)	0.61*** (0.53–0.69)	0.02 (0.03)	0.06* (0.03)
Constant	-	-	3.85	3.77
Observations	5,809	5,799	5,818	5,798
Adjusted *R*^2d^	0.02	0.02	0.05	0.05

Source: National Sample Survey of Physicians (2019), Association of American Medical Colleges

Note: *p < 0*.*05, **p < 0*.*01, ***p < 0*.*001, standard error, 95%*CI* are in parentheses. Weights were not applied in ordinal logistic regressions

^a^Emotional exhaustion, depersonalization models reported Odds Ratio (OR), OR = 1 is baseline reference, higher OR (OR > 1) is less desirable, indicated higher odds of burnout The odds ratios are reported odds of feeling burnout, callous everyday compared to other burnout, callous frequencies on the scale

bMetropolitan practice locations were derived from current practice location zip code, and cross walked to Rural–Urban Commuting Area codes (RUCA). We then classified each location into metropolitan area (RUCA 1–4) and non-metropolitan area (RUCA 5–10)

^c^Total number of children under 5 from our data is heavily right skewed, so we performed various types of transformations, but log transformation is not appropriate in our case due to high frequency of zeros

^d^The C statistics (pseudo *R*2) are 0.0187 and 0.023

For physicians with an academic affiliation, the odds of feeling emotional exhaustion every day were lower (OR 0.87; 95% CI 0.79–0.96; *P* < *0.001)* than for their non-affiliated peers, holding everything else constant. The odds of feeling depersonalization every day were also lower (OR 0.86; 95% CI: 0.78–0.95; *P* < *0.001)* than for non-affiliated peers. Female physicians reported experiencing higher emotional exhaustion and depersonalization than male physicians. Physicians working longer hours, or those working in primary care had higher odds of emotional exhaustion and feeling depersonalization. IMG status and greater work experience decreased both emotional exhaustion and depersonalization, which is consistent with previous research [16, 17].

Physicians with an academic affiliation had higher satisfaction on time use and career satisfaction (0.10 to 0.14 points, SE: 0.03, 0.02; *P* < 0.001) than non-affiliated peers. Male physicians, compared to female physicians, physicians married or in committed relationships, and those with greater work experience had higher satisfaction on both satisfaction measures. Physicians in the 35–55 age group and primary care reported lower satisfaction on both measures than the rest of the sample. We saw an increase in career satisfaction and time use satisfaction with every additional year in practice. IMG status was associated with higher satisfaction on both measures, which is consistent with previous research [17].

### Burnout, satisfaction, and faculty ranks

Table 3 presents results for faculty rank among academically affiliated with respect to physicians' emotional exhaustion, depersonalization, and satisfaction.

**Table 3:** Physician Burnout and Satisfaction, by Faculty Rank

	Ordinal Logistic Regressions		Multivariable Linear Regressions	
Independent Variables	Emotional Exhaustion^a^ (OR, 95% CI)	Depersonalization^a^ (OR, 95% CI)	Time Use Satisfaction (SE)	Career Satisfaction (SE)
Professor	Ref	Ref	Ref	Ref
Associate Professor	1.57*** (1.22–2.04)	1.44*** (1.11–1.88)	-0.09 (0.07)	-0.14** (0.06)
Assistant Professor	1.64*** (1.28–2.11)	1.22 (0.95–1.58)	-0.09 (0.07)	-0.14** (0.05)
Instructor	1.72*** (1.29–2.29)	1.35* (1.01–1.82)	-0.12 (0.08)	-0.23*** (0.06)
Other rankings	1.39 (0.91–2.12)	0.97 (0.63–1.49)	-0.18 (0.12)	-0.11 (0.09)
Weekly percent time spent teaching	0.99 (0.99–1.01)	0.99 (0.99–1.00)	0.00 (0.00)	0.00 (0.00)
Male	0.68*** (0.58–0.80)	0.80** (0.99–1.00)	0.16*** (0.05)	0.15*** (0.04)
Under-represented in Medicine (URM)	0.86 (0.58–1.28)	1.07 (0.72–1.60)	0.03 (0.10)	0.08 (0.08)
Asian	1.04 (0.70–1.53)	1.07 (0.72–1.58)	0.00 (0.10)	0.15 (0.08)
White	1.09 (0.76–1.57)	1.25 (0.87–1.81)	0.00 (0.09)	0.18* (0.08)
Heterosexual/ Straight	0.74 (0.52–1.05)	0.75 (0.52–1.09)	0.16 (0.10)	0.08 (0.08)
Age < 35	Ref	Ref	Ref	Ref
Age [35, 55)	1.22 (1.08–1.67)	0.98 (0.92–1.44)	-0.14 (0.07)	-0.02 (0.06)
Age [55,75)	1.27 (0.79–2.04)	0.68 (0.42–1.11)	-0.04 (0.14)	-0.07 (0.11)
Age ≥ 75	1.05 (0.44–2.53)	0.31* (0.11–0.86)	0.26 (0.25)	0.24 (0.19)
Rural	0.86 (0.70–1.06)	1.07 (0.87–1.33)	0.02 (0.06)	0.11* (0.05)
Practice location: Metropolitan^b^	1.44* (1.02–2.04)	1.20 (0.84–1.73)	-0.06 (0.04)	0.09 (0.08)
Married/Committed relationship	1.14 (0.92–1.41)	0.98 (0.79–1.22)	0.04 (0.06)	0.14*** (0.05)
Total number of children under 5^c^	0.78*** (0.67–0.92)	0.92 (0.77–1.09)	0.06 (0.05)	-0.01 (0.04)
Hours worked (per week)	1.01*** (1.01- 1.02)	1.00 (0.99–1.01)	-0.01*** (0.00)	0.00 (0.00)
Practice experience (years)	0.97*** (0.96–0.99	0.98*** (0.97–0.99)	0.00 (0.00)	0.01** (0.00)
Specialty: Medical Specialties	Ref	Ref	Ref	Ref
Specialty: Other	1.14 (0.93–1.42)	1.12 (0.91–1.40)	0.08 (0.06)	-0.02 (0.05)
Specialty: Primary Care	1.28* (1.05–1.58)	1.10 (1.90–1.36)	-0.07 (0.06)	-0.08 (0.05)
Specialty: Surgery	0.91 (0.73–1.14)	0.93 (0.75–1.18)	0.00 (0.06)	0.01 (0.05)
International Medical Graduates (IMGs)	0.64*** (0.51–0.76)	0.67*** (0.52–0.78)	0.07 (0.05)	0.03 (0.04)
Constant	-	-	3.84	3.64
Observations	2,342	2.338	2,348	2,344
Adjusted *R*^2d^	0.03	0.03	0.04	0.06

Source: National Sample Survey of Physicians (2019), Association of American Medical Colleges Notes: *p < 0*.*05, **p < 0*.*01, ***p < 0*.*001, standard error, 95%*CI* are in parentheses. Weights were not applied to ordinal logistic regressions

^a^Emotional exhaustion, depersonalization models reported Odds Ratio (OR), OR = 1 is baseline reference, higher OR (OR > 1) is less desirable, indicated higher odds of burnout The odds ratios are reported odds of feeling burnout, callous everyday compared to other burnout, callous frequencies on the scale

^b^Metropolitan practice locations were derived from current practice location zip code, and cross walked to Rural–Urban Commuting Area codes (RUCA). We then classified each location into metropolitan area (RUCA 1–4) and non-metropolitan area (RUCA 5–10)

^c^Total number of children under 5 from our data is heavily right skewed, so we performed various types of transformations, but log transformation is not appropriate in our case due to high frequency of zeros

^d^The C statistics (pseudo *R*2) are 0.025 and 0.026

Our ordinal logistic regression model showed, holding everything else constant, the odds of feeling emotional exhaustion every day for associate professors were higher (OR 1.57; 95% CI:1.22–2.04; *P* < *0.001*) than full-time professors; the odds of feeling emotional exhaustion every day were even higher for assistant professors (OR 1.64; 95% CI: 1.28–2.11; *P* < *0.0*01) and instructors (OR 1.72; 95% CI: 1.29–2.29; *P* < *0.001).* The odds of feeling depersonalization every day were higher for associate professors (OR 1.44; 95% CI: 1.11–1.88; *P* < *. 001*) than assistant professors (OR 1.22; 95% CI: 0.95–1.58; *P* = *0.12*)*,* and instructors (OR 1.35; 95% CI: 1.01–1.82; *P* = *0.05).* Daily emotional exhaustion and depersonalization odds decreased 1% with each 1% percent increase in teaching, although not statistically significant.

Assistant and associate professors reported significantly lower career satisfaction (*P* = 0.01) than full professors, and lower time use satisfaction, although the difference was not significant. Instructors also reported lower time use satisfaction and significantly lower career satisfaction (*P* < 0.001), compared to full professors.

## Discussion

Our research examines the relationship between academic affiliation and physicians’ burnout and satisfaction. We found that physicians with academic affiliations reported less burnout and higher satisfaction than those without academic affiliations. Furthermore, physicians ranked as full professor (a tenured position) reported less burnout and higher career-related satisfaction than those ranked at the lower levels of assistant or associate professors (both on tenure-track), or instructors (non-tenured positions.)

Existing research suggests both tangible and intangible benefits of academic affiliations. For example, academic affiliations may provide more research opportunities, access to role models, and increased mentorship opportunities [16, 18, 19]. Additionally, academically affiliated physicians may also have access to more diverse professional development opportunities than non-academic physicians. Moreover, Steinert et al. (2015) [20] found that many women physicians choose a career in academic medicine because of its potential for joy, fulfillment, and gratification, and because they find it interesting. Similarly, Yu et al. (2019) [21] showed that professional self-concept, or the perception of oneself as a member of the profession, protects medical school faculty against burnout. These feelings of professional self-concept are most likely higher for tenured professors, thus providing some explanation for the differences we saw among faculty ranks.

Tellingly, we found that daily emotional exhaustion and depersonalization decrease as percentage of time in teaching (weekly) increases. This may be explained by Shanafelt et al.’s (2009) article reporting that faculty who could spend more time on tasks they deemed ‘meaningful’ reported lower burnout than faculty who could not spend as much time on meaningful tasks [11]. Insofar as physicians generally find meaning and satisfaction in teaching [22–24] our findings suggest that teaching, specifically, may provide a protective buffer to burnout and boost satisfaction. Consistent with Shanafelt et al. (2009) [11], we found that higher-ranked faculty had the lowest burnout and highest satisfaction, as these physicians likely have the most control over their work activities.

However, even after controlling for time spent teaching, physicians with faculty ranks of associate professor, assistant professor and instructor still reported more emotional exhaustion and depersonalization than full professors. Although we did not find significant differences in our time use satisfaction variable across the ranks, assistant professors and instructors especially expressed less satisfaction on the career satisfaction variable. This is not particularly surprising for instructors, as they are not on a tenure track and therefore not yet strongly tied to the profession.

Among professors on the tenure track, assistant professors typically face the greatest promotion-related pressure, thereby explaining higher levels of burnout and lower career satisfaction.

It is important to note that our results differ slightly from Dandar et al.’s (2019) study of medical school faculty [7]. While Dandar et al. (2019) found that associate professors have higher burnout than assistant professors [7], we found assistant professors had higher levels of emotional exhaustion than associate professors (and lower depersonalization, although statistically not significant).We believe our conclusion is more credible due to the improvement in methods and more detailed burnout measures. Assistant professors are at the start of their tenure-track career and face the pressures and uncertainty of promotion and academic job security; therefore, it is logical that they would experience higher levels of burnout.

We found several demographic differences in emotional exhaustion, depersonalization, and career satisfaction. Consistent with previous research [19, 25, 26] we found that women physicians, physicians working longer hours, and those in primary care have higher odds of reporting burnout (both emotional exhaustion and depersonalization.) We found male physicians, physicians who are married or in a committed relationship, and those with longer work experience have higher satisfaction in both time use and career satisfaction. Additionally, we found that physicians who are married or partnered had both lower burnout and higher satisfaction. Further research examining demographic differences on these variables is needed to implement necessary practice and policy changes to enhance physician well-being. Finally, many of our preliminary findings, including connections between gender and IMG status, were outside the scope of our primary interest. However, we believe this is an important link that warrants further research and confirmation.

### Limitations

Our study is an important contribution to the literature on physician burnout and career satisfaction. However, it does have limitations. First, our regression results from a cross-sectional study do not suggest causal relationships. For example, physicians who choose to be in academic medicine may differ from their peers in unmeasured ways. There is a need to explore specific mechanisms, such as IMG status, enabling academic physicians to feel less burnt out and more satisfied with their careers. Second, it is essential to note that we analyzed data collected before the COVID-19 pandemic. A recent report from the National Institute for Health Care Management (NIHCM) shows an alarming increase in the percentage of healthcare workers burning out during the pandemic compared to pre-pandemic rates. More research is needed to assess the pandemic's short- and long-term impact on physician well-being and satisfaction [27].

## Conclusions

Our findings contribute to the literature on burnout and career satisfaction by exploring their association with academic affiliation and examining how they vary among different faculty ranks. An academic affiliation may be an essential factor in keeping physicians’ burnout levels lower and career satisfaction higher. This intimates that policies addressing physician well-being are not “one size fits all” and should consider factors such as academic affiliation, faculty rank and career stage, gender identity, the diversity of available professional opportunities, and institutional and social supports. For instance, department chairs and administrators in medical institutions could protect physicians’ time for academic activities like teaching to help keep burnout lower and career satisfaction higher.

## Supplementary Information

**Additional file 1**

## Data Availability

The datasets used and/or analyzed during the current study are available from all authors on reasonable request.

## Abbreviations

AAMC: Association of American Medical Colleges
AMA: American Medical Association
NSSP: National Sample Survey of Physicians
MBI: Maslach Burnout Inventory
IMG: International Medical Graduates
URM: Under-represented in Medicine
EE: Emotional Exhaustion
DP: Depersonalization
OR: Odds Ratio
NIHCM: National Institute for Health Care Management

## Footnotes

1 **Primary Care** consists of family medicine, general internal medicine, general pediatrics, and geriatric medicine. The **Medical Specialties** category consists of allergy and immunology, cardiology, critical care, dermatology, endocrinology, gastroenterology, hematology and oncology, infectious diseases, neonatal and perinatal medicine, nephrology, pulmonology, and rheumatology. **Surgical Specialties** consists of general surgery, colorectal surgery, neurological surgery, obstetrics and gynecology, ophthalmology, orthopedic surgery, otolaryngology, plastic surgery, thoracic surgery, urology, vascular surgery, and other surgical specialties. The **Other Specialties** category consists of anesthesiology, emergency medicine, neurology, pathology, physical medicine and rehabilitation, psychiatry, radiology, and all other specialties.

2 Weights were not applied in ordinal logistic regressions.

3 Academic affiliation in this paper indicates affiliation with a medical school only, differing from affiliation with an academic health center (AHC) or academic medical center (AMC). The latter was also measured in the survey as separate question, and about 76% (1824 out of 2389) of those who are affiliated with a medical school are also affiliated with an AHC or AMC.
