# Supplementary material for: Do physicians with academic affiliation have lower burnout and higher career-related satisfaction?
Source: BMC Med Educ. 2022 Apr 26;22:316. doi: 10.1186/s12909-022-03327-5 (PMC9039267; doi:10.1186/s12909-022-03327-5)
Supplement: Supplementary file 1 — Additional file 1 [file 12909_2022_3327_MOESM1_ESM.docx]

**Appendix**

## 1. Sampling method of the National Sample Survey of Physicians

Response rates on physician surveys, and health practitioner surveys more generally, have been declining (e.g., Weaver et al, 2019; Delnevo & Singh, 2019; Burt & Wordwell, 2006). Moreover, mail-based surveys cannot guarantee a minimum number of responses, making them cost prohibitive. Accordingly, we selected an online survey method that sampled from a panel of physicians: a subset of the total population of active, practicing physicians. We contracted with a firm (Toluna) which, by partnering with two others, was able to offer a large panel of physicians (N=86,951). The panel had been recruited by phone, after which physicians completed a double opt in via email and were verified against the AMA Physician database, considered to be the most comprehensive database of physicians in the US and used extensively in physician workforce research. A comparative analysis by physician age group, sex, and specialty group found the panel to be representative of the entire population of active, practicing physicians in the U.S.

Power calculations based on needing separate estimates for combinations of physician age group, sex, and specialty group set the minimum sample size for the survey at 3,000. To improve statistical power beyond the minimum needed, a sample size of 6,000 was set. Our sampling method further set minimum numbers of responses we needed for each of twenty-four age-sex- specialty sampling strata based on the representation of each stratum within the study population, with an additional stratum for all physicians to allow for sample variability. For statistical power purposes, we upward adjusted two strata quotas (female surgeons 60 and over and female medical specialists 60 and over). All panelists (N=86,951) were invited to participate in the survey and responses were accepted until all strata minimum sample sizes had been met and a total of n=6,000 had been achieved.

This resulted in a sampling that does not yield a standard response rate because once we had achieved our strata minimum sample sizes and total n (6,000), we stopped accepting responses. Moreover, once all physician’s strata had filled, respondents in age-sex-specialty strata that were also complete were rejected prior to achieving our total of n=6,000. Thus, while 6,000 responses out of 86,951 yields a “response rate” of 7%, we know that 8,045 potential

respondents began the survey but were disqualified (largely because their strata were full, though 225 were disqualified because we determined they were residents or fellows, who are physicians but out of scope for our study). Had we accepted all those responses, our “response rate” would have been 16%. We cannot know how many additional physicians would have responded if we had not closed the survey once we achieved 6,000 qualifying responses, leaving a final “response rate” incalculable.

We do know that the sample we drew met our statistical power needs, and we know that those responses were drawn from a panel that is representative of the population. Additionally, our stratified quota sample allows us larger samples of smaller sub-populations (e.g., female surgeons 60 and over) than we would have achieved with a strict random sample. We also ran a post-survey analysis to assess the representativeness of our final sample of respondents and found it to be representative on all our initial selection variables of age group, specialty group, and sex (see Table A). Our post-survey analysis did find that international medical graduates (IMGs) were under-represented in our sample. A set of analytical weights was created with a combination of cell weighting (for combinations of age group, sex, and specialty group) and rim weighting (for IMGs). The final weighted data thus match study population values for age group, sex and specialty group (combined) and for IMGs (in total). Weighting targets were based on the AMA Physician Characteristics Database.

Table 1: Comparison of Sample and Population Distributions across Key Variables

| **Specialty group** | **Sex** | **Age group** | **Percent of unweighte d sample** | **Percent of weighted sample** | **Percent of population of active, practicing physicians in the U.S. (based on AMA Masterfile data)** |
| --- | --- | --- | --- | --- | --- |
| Medical Specialties | F | Under age 50 | 2.9% | 3.3% | 3.3% |
| Medical Specialties | F | 50-59 | 1.1% | 1.1% | 1.1% |
| Medical Specialties | F | 60 and over | 0.7% | 0.9% | 0.9% |
| Medical Specialties | M | Under age 50 | 6.1% | 4.3% | 4.3% |
| Medical Specialties | M | 50-59 | 3.9% | 2.8% | 2.8% |

| Medical Specialties | M | 60 and over | 5.2% | 4.3% | 4.3% |
| --- | --- | --- | --- | --- | --- |
| Other | F | Under age 50 | 3.2% | 4.9% | 4.9% |
| Other | F | 50-59 | 1.5% | 2.4% | 2.4% |
| Other | F | 60 and over | 1.3% | 2.3% | 2.3% |
| Other | M | Under age 50 | 7.2% | 7.4% | 7.4% |
| Other | M | 50-59 | 4.0% | 5.1% | 5.1% |
| Other | M | 60 and over | 4.4% | 7.5% | 7.6% |
| Primary Care | F | Under age 50 | 7.9% | 8.4% | 8.4% |
| Primary Care | F | 50-59 | 4.4% | 3.9% | 3.9% |
| Primary Care | F | 60 and over | 3.1% | 3.0% | 3.0% |
| Primary Care | M | Under age 50 | 9.5% | 6.6% | 6.6% |
| Primary Care | M | 50-59 | 5.7% | 5.0% | 5.0% |
| Primary Care | M | 60 and over | 9.4% | 7.8% | 7.9% |
| Surgery | F | Under age 50 | 2.4% | 3.0% | 3.0% |
| Surgery | F | 50-59 | 1.2% | 1.2% | 1.2% |
| Surgery | F | 60 and over | 0.6% | 0.9% | 0.8% |
| Surgery | M | Under age 50 | 5.1% | 4.6% | 4.7% |
| Surgery | M | 50-59 | 4.6% | 3.7% | 3.7% |
| Surgery | M | 60 and over | 4.8% | 5.5% | 5.5% |

Sources: National Sample Survey of Physicians, 2019, Association of American Medical Colleges; AMA Physician Characteristics database, 2018.

References

1. Weaver, Leslie; Beebe, Timothy J.; and Todd Rockwood. 2019. “The impact of survey mode on the response rate in a survey of the factors that influence Minnesota physicians disclosure practices.” BMC Medical Research Methodology. 19 (73). <https://doi.org/10.1186/s12874-019-0719-7>
2. Delnevo, Cristine D.; and Binu Singh. 2019. “The effect of a web-push survey on physician survey response rates: a randomized experiment." Research Square. Posted 7/31/2019. https://doi.org/10.21203/rs.2.12242/v1
3. Catharine W. Burt; and David Woodwell. 2006. “Tests of methods to improve response to physician surveys.” Centers for Disease Control and Prevention, National Center for Health Statistics, Division of Health Care Statistics. Accessed: 5/21/20. <https://nces.ed.gov/FCSM/pdf/2005FCSM_Burt_Woodwell_VIIB.pdf>

Figure 1: Flow Chart for Methods


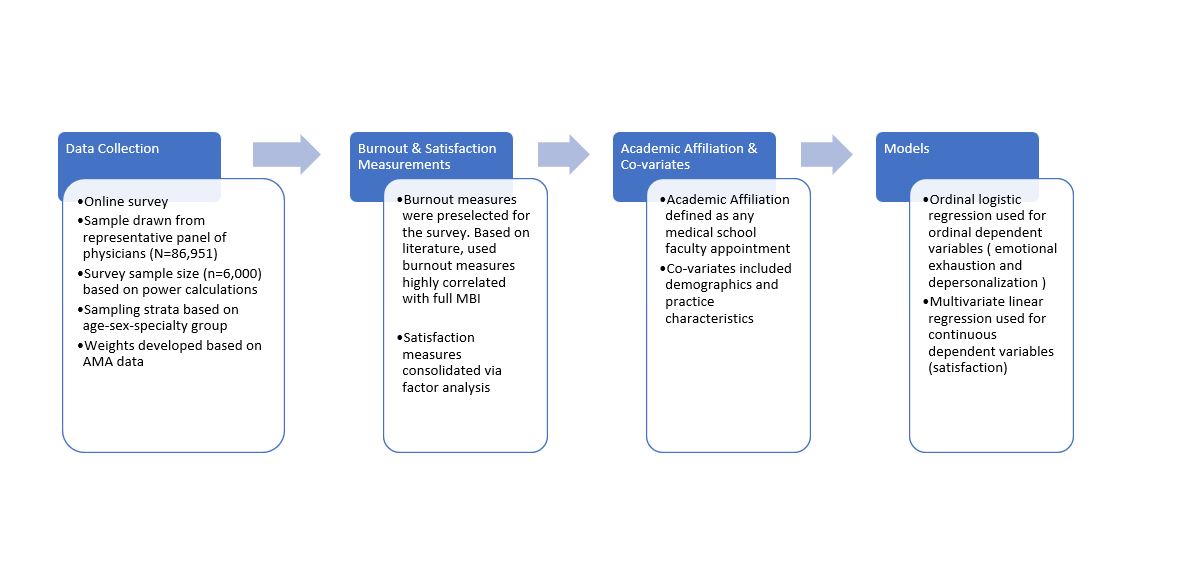


Source: National Sample Survey of Physicians (2019), Association of American Medical Colleges

| Table 2. Summary Statistics | | | |
| --- | --- | --- | --- |
| **Demographic, family structure and work-related characteristics** | Physicians  (n= 6,000) | Academic Affiliates  (n= 2,406) | Non-Academic Affiliates  (n= 3,572) |
| **Gender (%)** |  |  |  |
| Male | 65% | 65% | 64% |
| Female | 35% | 35% | 36% |
| **Sexual Orientation (%)** |  |  |  |
| Heterosexual | 96% | 96% | 96% |
| **Age (yrs.)**  Mean (SD) | 52  (11.3) | 52  (11.3) | 52  (11.3) |
| **Race and Ethnicity (%)** |  |  |  |
| American Indian / Alaska Native | 0.4% | 0.5% | 0.3% |
| Asian | 23% | 20% | 24% |
| Native Hawaiian/Pacific Islander | 0.4% | 0.5% | 0.4% |
| White | 69% | 72% | 67% |
| Latino | 3.7% | 3.7% | 3.8% |
| Other | 3.2% | 3.3% | 3.1% |
| **Rurality of place grew up (%)** |  |  |  |
| Rural | 14% | 14% | 14% |
| Suburban | 56% | 54% | 57% |
| Urban | 30% | 32% | 28% |
| Military and Gov. | 0% | 0% | 1% |
| **Practice location (%)** |  |  |  |
| Metropolitan | 96% | 95% | 93% |
| **Marital status (%)** |  |  |  |
| Married/Partnered | 84% | 85% | 83% |
| Non-married/Non- partnered | 6% | 5% | 7% |
| **Total number of children under 5**  Mean (SD) | 0  (0.5) | 0  (0.5) | 0  (0.5) |
| **Work Hours (per week)**  Mean (SD) | 47  (16.0) | 49  (16.0) | 46  (15.9) |
| **Experience (yrs.)**  Mean (SD) | 20  (11.5) | 19  (11.6) | 20  (11.4) |
| **Specialties (%)** |  |  |  |
| Medical Specialties | 17% | 20% | 15% |
| Other | 30% | 31% | 28% |
| Primary Care | 35% | 28% | 39% |
| Surgery | 18% | 21% | 18% |
| **Medical Graduates (%)** |  |  |  |
| International Medical Graduates (IMGs) | 27% | 24% | 29% |
| United States Medical Graduates (USMG) | 73% | 76% | 71% |
| **Teaching (%)**  Mean (SD) | 2  (6.7) | 5  (8.6) | 1  (4.3) |

Source: National Sample Survey of Physicians (2019), Association of American Medical Colleges

| \| Table 3. Mean Score of Emotional Exhaustion, Depersonalization, Satisfaction Measures by Academic Affiliation Status and Faculty Ranks \| \| --- \| | | | | | |
| --- | --- | --- | --- | --- | --- | --- |
| Descriptive | | Mean score of Emotional Exhaustion | Mean score of depersonalization | Mean score of  Time Use  Satisfaction | Mean score of Career Satisfaction |
|  |  | **(SD)** | **(SD)** | **(SD)** | **(SD)** |
| Faculty  Appointment | **Non-faculty**  **(n=3,572)** | 3.22 | 2.59 | 3.51 | 4.00 |
|  |  | (1.51) | (1.50) | (1.10) | (0.89) |
|  | **Faculty**  **(n=2,406)** | 3.14 | 2.50 | 3.58 | 4.15 |
|  |  | (1.45) | (1.49) | (0.99) | (0.80) |
|  |  |  |  |  |  |
|  |  |  |  |  |  |
| Faculty Ranking | **Professor**  **(n=316)** | 2.65 | 2.11 | 3.73 | 4.41 |
|  |  | (1.38) | (1.40) | (0.98) | (0.70) |
|  | **Associate**  **Professor**  **(n=638)** | 3.17 | 2.61 | 3.55 | 4.15 |
|  |  | (1.46) | (1.53) | (0.99) | (0.81) |
|  |  |  |  |  |  |
|  | **Assistant**  **Professor**  **(n=974)** | 3.23 | 2.52 | 3.57 | 4.11 |
|  |  | (1.44) | (1.46) | (0.98) | (0.79) |
|  |  |  |  |  |  |
|  | **Instructor**  **(n=379)** | 3.27 | 2.64 | 3.54 | 4.05 |
|  |  | (1.46) | (1.54) | (1.02) | (0.82) |
|  | **Other**  **rankings**  **(n=99)** | 3.00 | 2.27 | 3.53 | 4.22 |
|  |  | (1.43) | (1.50) | (0.98) | (0.87) |
|  |  |  |  |  |  |

Source: National Sample Survey of Physicians (2019), Association of American Medical College

Figure 2. Factor Loading Results for Satisfaction Measures

Source: National Sample Survey of Physicians (2019), Association of American Medical Colleges

Figure 3. Odds Ratios for Physician Burnout Controlling for Demographic and Practice Characteristics (Coefficient Plot)

Source: National Sample Survey of Physicians (2019), Association of American Medical Colleges

Figure 4. Odds Ratios for Physician Burnout by Faculty Rank (Coefficient Plot)

Source: National Sample Survey of Physicians (2019), Association of American Medical Colleges
